# MiR-29 Regulates *de novo* Lipogenesis in the Liver and Circulating Triglyceride Levels in a Sirt1-Dependent Manner

**DOI:** 10.3389/fphys.2019.01367

**Published:** 2019-10-29

**Authors:** Yu-Han Hung, Matt Kanke, Catherine Lisa Kurtz, Rebecca L. Cubitt, Rodica P. Bunaciu, Liye Zhou, Phillip J. White, Kasey C. Vickers, Mohammed Mahmood Hussain, Xiaoling Li, Praveen Sethupathy

**Affiliations:** ^1^Department of Biomedical Sciences, Cornell University, Ithaca, NY, United States; ^2^Department of Genetics, The University of North Carolina at Chapel Hill, Chapel Hill, NC, United States; ^3^Diabetes and Obesity Research Center, NYU Winthrop Hospital, Mineola, NY, United States; ^4^Duke Molecular Physiology Institute, Duke University, Durham, NC, United States; ^5^Department of Medicine, Vanderbilt University, Nashville, TN, United States; ^6^Laboratory of Signal Transduction, National Institute of Environmental Health Sciences, Durham, NC, United States

**Keywords:** microRNA (miRNA), miR-29, liver, lipogenesis, Sirtuin 1 (Sirt1)

## Abstract

MicroRNAs (miRNAs) are known regulators of lipid homeostasis. We recently demonstrated that miR-29 controls the levels of circulating cholesterol and triglycerides, but the mechanisms remained unknown. In the present study, we demonstrated that systemic delivery of locked nucleic acid inhibitor of miR-29 (LNA29) through subcutaneous injection effectively suppresses hepatic expression of miR-29 and dampens *de novo* lipogenesis (DNL) in the liver of chow-fed mice. Next, we used mice with liver-specific deletion of Sirtuin 1 (L-*Sirt1* KO), a validated target of miR-29, and demonstrated that the LNA29-induced reduction of circulating triglycerides, but not cholesterol, is dependent on hepatic Sirt1. Moreover, lipidomics analysis revealed that LNA29 suppresses hepatic triglyceride levels in a liver-Sirt1 dependent manner. A comparative transcriptomic study of liver tissue from LNA29-treated wild-type/floxed and L-*Sirt1* KO mice identified the top candidate lipogenic genes and hepatokines through which LNA29 may confer its effects on triglyceride levels. The transcriptomic analysis also showed that fatty acid oxidation (FAO) genes respond differently to LNA29 depending on the presence of hepatic Sirt1. Overall, this study demonstrates the beneficial effects of LNA29 on DNL and circulating lipid levels. In addition, it provides mechanistic insight that decouples the effect of LNA29 on circulating triglycerides from that of circulating cholesterol.

## Introduction

The liver is the primary site of lipid synthesis and is a major source of circulating lipids (Nguyen et al., 2008; Tiwari and Siddiqi, 2012). Dysregulation of hepatic lipid metabolism is involved in the pathogenesis of many metabolic disorders. For example, abnormal lipid accumulation in the liver is the hallmark of fatty liver disease (Matsuzaka et al., 2007; Dorn et al., 2010) and exacerbates insulin resistance (Banerji et al., 1995; Bajaj et al., 2003). Elevated lipid synthesis and secretion by the liver can cause hyperlipidemia, which is a significant risk factor for atherosclerosis and cardiovascular disease (Zoltowska et al., 2001; Adiels et al., 2008). Given the critical contribution of hepatic lipid metabolism to various metabolic diseases and related complications, there have been numerous efforts to identify key regulators of lipid levels (Singh et al., 2017; Townsend and Newsome, 2017), as these represent candidate therapeutic targets.

Accumulating evidence suggests that microRNAs (miRNAs) are key post-transcriptional regulators of lipid homeostasis (Moore, 2013; Vickers et al., 2013a). We and others have demonstrated that miRNAs play an important role in regulating lipid metabolism in the liver. For example, we identified that hepatic miR-27b acts as a master regulator of lipid balance through modulation of genes encoding key lipogenic enzymes such as *Gpam* (Vickers et al., 2013b). Another miRNA in the liver, miR-30c, is involved in the control of lipoprotein secretion by targeting *Mttp* (Soh et al., 2013). In addition, we and others demonstrated that hepatic miR-223 coordinates cholesterol biosynthesis, uptake and efflux in the liver in part through regulation of *Abca1* (Vickers et al., 2014). Additional studies have identified other miRNAs that are involved in different aspects of lipid regulation in the liver, including but not limited to miR-34a (Choi et al., 2013), miR-33a/b (Najafi-Shoushtari et al., 2010; Rayner et al., 2011), miR-122 (Esau et al., 2006; Cheung et al., 2008), miR-192 (Lin et al., 2017), and miR-148a (Cheng et al., 2017; Heo et al., 2019), several of which have been shown to be dysregulated in fatty liver disease and hyperlipidemia (Jeon and Osborne, 2016).

Recently, we uncovered the role of another miRNA, miR-29, in regulating lipid homeostasis. First we demonstrated that miR-29 is significantly elevated in the livers of mice fed an obesogenic high-fat diet as well as in Zucker Diabetic Fatty (*fa/fa*) rats, and may be involved in fine-tuning the liver FOXA2 transcriptional network (Kurtz et al., 2014). Later we showed using locked nucleic acid (LNA) inhibitors (Veedu and Wengel, 2010) that 1-week, acute suppression of miR-29 (LNA29) *in vivo* effectively reduces hepatic miR-29 levels and leads to significant reduction of circulating triglyceride and cholesterol in adult chow-fed mice (Kurtz et al., 2015). We postulated in that study that the lipid-lowering effect induced by LNA29 may be in part due to elevated Sirtuin 1 (*Sirt1*), which is a direct target of miR-29 (Kurtz et al., 2015). However, the mechanisms underpinning the effects of LNA29 were not fully understood. In the present study, we demonstrate that acute suppression of miR-29 dampens hepatic *de novo* lipogenesis (DNL) in the mouse liver and show that Sirt1 mediates the effects of miR-29 inhibition on circulating triglycerides but not cholesterol.

## Materials and Methods

### Animals

Mouse studies – The mice enrolled in the *in vivo* LNA29 studies were maintained on a 12 h light/dark cycle with access to diet and water *ad libitum*. To evaluate the acute effects of LNA29 on lipid homeostasis, C57BL/6J mice (female 9–11 weeks old) on chow-diet (Lab Diet 5047) were used. To evaluate the role of hepatic Sirt1 in mediating the effects of LNA29 on lipid homeostasis, the mouse model of liver-specific knockout of Sirt1 and floxed control littermates (female 15–20 week old mice) with C57BL/6J background were used (Purushotham et al., 2009). In these studies, the blood and the liver tissues collected during the fed state were used for plasma metabolite analysis, RNA-seq analysis, and lipidomics. In addition, liver tissue from a subset of mice were collected from the fasted state as the standard procedure for *de novo* lipid synthesis assay, in which the tissues are incubated with radiolabeled acetate substrates *ex vivo*.

Rat studies – The rats included in the studies were maintained on a 12 h light/dark cycle with access to diet and water *ad libitum*. To evaluate the association of hepatic miR-29 levels and expressions of lipogenesis genes, 5.5 month-old male *fa/fa* Zucker Fatty rats (ZFRs) and control Zucker Lean rats (ZLRs) fed on chow diet were used. To study the hepatic genes in response to 3,6-dichlorobenzo[b]thiophene-2-carboxylic acid (BT2) in the ZFR model, 3 month-old male ZFR received daily intraperitoneal injections of BT2 (20 mg/kg; dissolved in sterile dimethylsulfoxide) or vehicle (equal volume of sterile dimethylsulfoxide) for 1 week. The injections were performed daily at Zeitgeber time (ZT) 3. More details of BT2 treatments are described in White et al. (2018). Liver tissue was collected during fed state for RNA-seq analysis.

All animal procedures were performed with the approval and authorization of the Institutional Animal Care and Use Committee (IACUC) at each participating institution.

### *In vivo* LNA Study

Animals received one subcutaneous injection of RNase-free sterile saline (Bio Scientific, Austin, TX, United States) or LNAs against mmu-miR-29a-3p (5′-ATTTCAGATGGTGCT-3′) and mmu-miR-29bc-3p (5′-ATTTCAAATGGTGCT-3′) at 20 mg/kg each (Qiagen) at ZT 3. We have demonstrated previously that one subcutaneous injection of LNA against miR-29 with this concentration effectively suppresses hepatic miR-29 expressions (Kurtz et al., 2015). We have also demonstrated in a previously published study that saline and LNA-scramble sequence (LNA-scr) exert similar effects on our physiological parameters of interest and therefore both can serve as control treatment for the LNA-29 *in vivo* study (Hung et al., 2019). We therefore used saline as control treatment in the present study. For radiolabeled assays of *de novo* lipid synthesis, fresh liver was collected from overnight fasted animals at day 7 post-dose of LNA. For plasma metabolite and transcriptome analysis, blood and liver were collected from non-fasted animals at day 7 post-dose of LNA. The *in vivo* LNA study was performed at National Health Institutes as well as at NYU Winthrop Hospital. No significant differences were observed in body weights at day 7 post-dose between saline and LNA29 treatment. All animal procedures were performed with the approval and authorization of the IACUC at each participating institution.

### Plasma Metabolite Analysis

Plasma was prepared by spinning whole blood for 10 min at 10,000 × *g*, 4°C. Plasma was used to measure circulating cholesterol and triglyceride at the UNC Nutrition Obesity Research Center (NORC) Nutritional Biochemistry and Molecular Biology core facility.

### *De novo* Lipid Synthesis Assay

3H-acetate was purchased from NEN Life Science Products. Mice were fasted overnight (16 h) before anesthesia. 1 ml of serum free DMEM containing 100 uCi of 3H-acetate was added to each well in 12-well plate and pre-warmed at 37°C incubator for 20 min. About 50 mg of fresh liver pieces were weighed and incubated with isotope containing medium for 3 h. The wet weight was used for normalization later. After incubation, tissues were washed three times with PBS, chopped into fine pieces, and placed into 750 μl of chloroform: methanol (1:2) solution overnight. The next day, 250 μl of chloroform was added to each tube and incubated in room temperature for 15 min. 250 μl of distilled water was added to each tube and the tubes were centrifuged for 10 min at 12,000 × *g*. The bottom layer was taken and evaporated overnight. Total lipids were resuspended in 100 μl of chloroform. Different lipids were separated on silica-60 thin layer chromatography (TLC) plates (Hexane: diethyl ether: glacial acetic acid = 82:17:2). Pure TG, cholesterol, and phospholipid were used as markers on TLC plate. The plates were exposed to iodine to visualize bands. The bands containing TG, cholesterol, or phospholipid were scraped off from the plates and counted in a scintillation counter.

### RNA Extraction and Real-Time qPCR

Total RNA was isolated using the Total RNA Purification kit (Norgen Biotek, Thorold, ON, Canada). High Capacity RNA to cDNA kit (Life Technologies, Grand Island, NY, United States) was used for reverse transcription of RNA. TaqMan microRNA Reverse Transcription kit (Life Technologies) was used for reverse transcription of miRNA. Both miRNA and gene expression qPCR were performed using TaqMan assays (Life Technologies) with either TaqMan Universal PCR Master Mix (miRNA qPCR) or TaqMan Gene Expression Master Mix (mRNA qPCR) per the manufacturer’s protocol, on a BioRad CFX96 Touch Real Time PCR Detection System (Bio-Rad Laboratories, Richmond, CA, United States). Reactions were performed in triplicate using either U6 (miRNA qPCR) and Rps9 (mRNA qPCR) as the normalizer.

### RNA Library Preparation and Sequencing

RNA-sequencing libraries of the liver were prepared using the Illumina TruSeq polyA + Sample Prep Kit. Sequencing of mouse livers harvested during fed state was carried out on the Illumina HiSeq2000 platform with single-end 100 bp sequencing depth at the UNC High Throughput Sequencing Core Facility. Sequencing of ZFR livers harvested during fed state was performed using the same sequencing platform and parameters at the Cornell RNA-sequencing core facility. Raw sequencing data as well as normalized counts are available through GEO accession GSE129539.

### Bioinformatics Analysis

The sequencing reads were aligned to mouse genome (mm10) for mouse samples and rat genome (rn6) for rat samples. RNA-sequencing reads were mapped to genome release using STAR (v2.5.3a) (Dobin et al., 2013) and transcript quantification was performed using Salmon (v0.6.0) (Patro et al., 2017). Differential gene expression analysis was accomplished using DESeq2 (Love et al., 2014). The Benjamini–Hochberg method was used for multiple testing correction. One sample in the group of floxed mice receiving saline treatment was considered as an outlier based on the PCA analysis and thereby excluded from the entire RNA-seq analyses. MiRNA binding site enrichment among differentially expressed genes was determined using miRhub (Baran-Gale et al., 2013). Gene pathway analyses with specific subsets of genes were performed using Enrichr (Chen et al., 2013; Kuleshov et al., 2016) and the results were shown using WikiPathways’s cell signaling pathway database.

### Hepatic Lipid Extraction for Lipidomics

Snap-frozen liver tissues (10 mg) were homogenized in 300 μL chilled 50% methanol (Optima^TM^ LC/MS Grade, A456-500, Fisher Chemical) diluted in HPLC grade water (Fisher Chemical, W5-1). 600 μL of methylene chloride (Optima^TM^ LC/MS Grade, D151-1, Fisher Chemical) was added to the homogenates and vortexed for 10 s. 300 μL HPLC grade water was then added and vortexed for 10 s. The tissue homogenates were centrifuged at 13,000 rpm for 15 min at 4°C. The bottom organic layer was collected into silica tubes and the supernatants were air dried by speed vacuum. Samples were redissolved in 150 μL of isopropanol (IPA)/acetonitrile (ACN)/H2O (65:30:5) prior to analysis. 25 μg/mL of TG (15:15:15) were added as internal standard prior to analysis and used for normalization.

### Liquid Chromatography and Mass Spectrometry

Chromatographic separation was performed on a Vanquish UHPLC system with an Accucore C30, 2.6 μm column (2.1 mm id × 150 mm) coupled to a Q Exactive^TM^ Hybrid Quadrupole-Orbitrap High Resolution Mass Spectrometer (Thermo Fisher Scientific, San Jose, CA, United States). The mobile phase consisted of solvent A containing 60% ACN, 40% H2O, 10 mM Ammonium Formate with 0.1% Formic Acid and solvent B containing 90% IPA, 10% ACN, 10 mM Ammonium Formate with 0.1% Formic Acid. The gradient was as follows: 0–1.5 min, 32% solvent B; 1.5–4 min, 32–45% solvent B; 4–5 min, 45–52% solvent B; 5–8 min 52–58% solvent B; 8–11 min, 58–66% solvent B; 11–14 min, 66–70% solvent B; 14–18 min, 70–75% solvent B; 21–25 min, isocratic 97% solvent B, 25–25.1 min 97–32% solvent B; followed by 4 min of re-equilibration of the column before the next run. The flow rate was 260 μl/min and the Injection volumes were set to 2 μL. To avoid possible bias, the sequence of injections was randomized. All of the samples were analyzed by negative electrospray ionization in data-dependent MS-MS mode, Nitrogen as sheath, auxiliary, and sweep gas was set at 50, 5, and 1 U, respectively. Other conditions included the following: resolution, 1,20,000 full width at half maximum; automatic gain control target, 3e6 ions; maximum injection time, 100 ms; scan range, 67–1000 m/z; spray voltage, 3.50 kV; and capillary temperature, 275°C. Data-dependent MS-MS spectra were generated through the use of the following conditions: resolution, 15,000 full width at half maximum; automatic gain control target, 1e5 ions; maximum injection time, 50 ms; isolation window, 0.4 m/z.

### Lipidomics Data Analysis

The acquired data set was processed using Thermo Scientific LipidSearch^TM^ software version 4.1 with the following workflow. First, the individual data files were searched for product ion MS/MS spectra of lipid precursor ions. MS/MS fragment ions were predicted for all precursor adduct ions measured within ±5 ppm. The product ions that matched the predicted fragment ions within a ±5 ppm mass tolerance were used to calculate a match-score, and those candidates providing the highest quality match were determined. Next, the search results from the individual positive ion files from each sample group were aligned within a retention time window (±0.1 min) and the data were merged for each annotated lipid. The annotated lipids that were not ionized by NH4+ were then filtered to reduce false positives. The resulting data of TG (with [M + NH4]^+^ ion) was analyzed using MetaboAnalyst (Chong and Xia, 2018; Chong et al., 2018). Specifically, the data was normalized by tissue weight followed by log transformation and Pareto scaling. Two-tailed Student’s t tests were performed to calculate raw *P*-values and adjusted *P*-values (FDR).

### Statistics

The quantitative data are reported as an average of biological replicates ± standard error (SE) of the mean. Statistical differences were assessed by two-tailed Student’s *t*-test with threshold *P*-value <0.05. For the experiments that were performed previously and repeated in the present study, the statistical differences were assessed by one-tailed Student’s *t*-test with threshold *P*-value <0.05. Given that we only had *n* = 2 for the group of floxed mice receiving saline treatment and that there is no significant difference between WT and floxed mice in circulating cholesterol (WT 95 ± 10 mg/dL; floxed 105 ± 5 mg/dL) and TG (WT 86 ± 15 mg/dL; floxed 92 ± 19 mg/dL), we pooled WT and floxed mice into one group for meaningful statistical comparisons.

## Results

### Acute Systemic Suppression of miR-29 Dampens *de novo* Lipid Synthesis in the Liver and Lowers Circulating Lipid Levels

**FIGURE 1:**
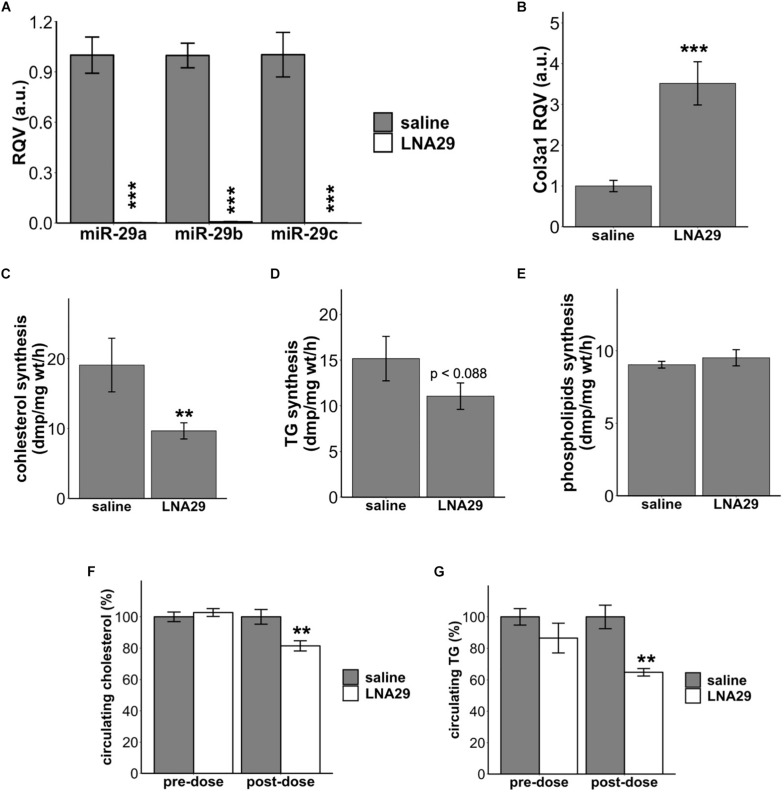
Acute treatment of LNA29 inhibits *de novo* lipid synthesis in the liver and lowers circulating cholesterol and triglyceride (TG) in chow-fed wild type (WT) mice. **(A)** RT-qPCR of hepatic miR-29 family of chow-fed WT mice with saline or LNA29 treatment. LNA29 leads to 444, 126, 614 fold suppression in miR-29a, b, and c, respectively. **(B)** qPCR of *Col3a1*, a validated miR-29 target gene, of chow-fed WT mice with saline or LNA29 treatment. **(C)** 3H-acetate incorporation into cholesterol with liver samples harvested from saline- or LNA29-treated WT mice. **(D)** 3H-acetate incorporation into TG with liver samples harvested from saline- or LNA29-treated WT mice. **(E)** 3H-acetate incorporation into phospholipids with liver samples harvested from saline- or LNA29-treated WT mice. **(F)** Non-fasted circulating cholesterol of mice with saline or LNA29 treatment. **(G)** Non-fasted circulating TG of mice with saline or LNA29 treatment. Given that the circulating data presented in this figure is a repeat of our previous findings, the one-tailed Student’s *t*-test was used. ^∗∗^*P* < 0.01, ^∗∗∗^*P* < 0.001 by one-tailed Students’ *t* test (saline, *n* = 6; LNA29, *n* = 6).

We have previously demonstrated that one dose of LNA29 injection (20 mg/kg for LNA29a and LNA29b/c each) in chow-fed C57BL/6J mice significantly lowers circulating triglyceride (TG) and cholesterol levels compared to saline treatment (Kurtz et al., 2015). We have also previously reported that LNA-mediated suppression of the miR-29 family leads to hepatic transcriptome profiles that indicate suppression of cholesterol and fatty acid synthesis pathways (Kurtz et al., 2015). However, whether LNA29 treatment in mice suppresses hepatic lipid synthesis *in vivo* has not yet been examined. To address this, we first repeated the LNA29 experiment, in which the chow-fed C57BL/6J mice received one subcutaneous injection of either LNA29 or saline control. Fresh liver tissues were collected from fasted mice at day 7 post-dose for DNL assays. We found that LNA29 treatment significantly suppresses the levels of hepatic miR-29 family members (miR-29a, b and c exhibit 444-, 126-, and 614-fold suppression, respectively) and significantly elevates a validated miR-29 target gene *Col3a1* compared to saline treatment as determined by qPCR (Figures 1A,B). We showed that LNA29 moderately decreases 3H-acetate incorporation into cholesterol as well as TG but has no effects on phospholipids compared to saline control (Figures 1C–E). Also, as shown previously (Kurtz et al., 2015), non-fasted circulating levels of TG and cholesterol were significantly lowered by LNA29 compared to saline treatment (Figures 1F,G). These findings overall suggest that the moderate reduction of hepatic DNL may partially contribute to the significant reduction of circulating lipids upon treatment with LNA29. Note that treatment with LNA inhibitors of another liver-expressed miRNA, miR-455, has little effects on liver lipids (Supplementary Figures S1A–C), which demonstrates that the effects of LNA29 on circulating lipids and DNL does not represent a generic effect of LNAs but rather a specific effect of suppressing miR-29.

### The Reduction of Circulating TG by LNA29 Is Abolished in the Absence of Liver Sirt1

**FIGURE 2:**
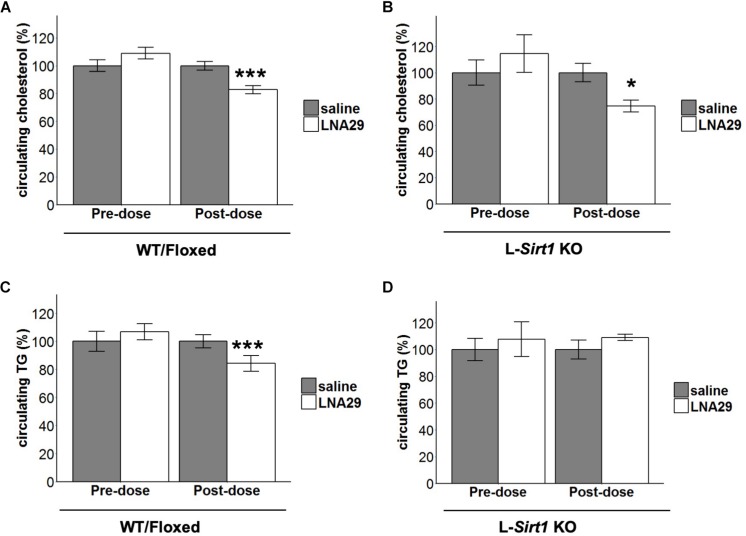
The absence of hepatic Sirt1 blunts LNA29-induced reduction of circulating triglyceride (TG). **(A)** Non-fasted circulating cholesterol of WT/Floxed mice with saline or LNA29 treatment. **(B)** Non-fasted circulating cholesterol of L-*Sirt1* KO mice with saline or LNA29 treatment. **(C)** Non-fasted circulating TG of WT/Floxed mice with saline or LNA29 treatment. **(D)** Non-fasted circulating TG of L-*Sirt1* KO mice with saline or LNA29 treatment. Data of WT/Floxed mice in response to LNA29 was assessed by one-tailed Student’s *t*-test (Floxed/WT, *n* = 11–12/treatment group) as the data is a validation of our previous findings. The data of L-Sirt1 KO mice in response to LNA29 was assessed by two-tailed Student’s *t*-test (L-Sirt1 KO, *n* = 5–6/treatment group). ^∗^*P* < 0.05, ^∗∗∗^*P* < 0.001.

We have previously validated that Sirtuin-1 (*Sirt1*) is a miR-29 target gene and that protein levels of hepatic Sirt1 are increased by LNA29 treatment in mice (Kurtz et al., 2015). Given that Sirt1 is known to be involved in the regulation of lipid metabolism in the liver (Purushotham et al., 2009; Yin et al., 2014) and that its expression is post-transcriptionally regulated by miR-29 (Bae et al., 2014), we hypothesized that the LNA29-induced lipid lowering effects are in part mediated by loss of miR-29 regulation of Sirt1 in the liver. To test this hypothesis, we employed a liver-specific Sirt1 knockout (L-*Sirt1* KO) mouse model (Purushotham et al., 2009). A set of L-*Sirt1* KO mice and control mice, including both C57BL/6J wild type and floxed (WT/Floxed) mice, were treated with LNA29 or saline and examined at day 7 post-dose (Supplementary Figure S2A). In the absence of hepatic Sirt1, LNA29 was still able to effectively suppress the miR-29 family and increase its target gene *Col1a1* (Supplementary Figures S2B,C). We found that non-fasted circulating levels of cholesterol are significantly lowered by LNA29 in both L-*Sirt1* KO and WT/Floxed mice (Figures 2A,B; by Student’s *t*-test). Notably, however, we found that non-fasted circulating levels of TG are significantly lowered by LNA29 only in WT/Floxed mice, and not in L-*Sirt1* KO mice (Figures 2C,D; by Student’s *t*-test). A two-way ANOVA test was also applied to examine the effect of treatment type (saline vs. LNA29), genotype (WT/floxed vs. KO), and the interaction on the circulating cholesterol and TG. Consistent with the conclusions made by Student’s *t*-test, while the treatment effect is significant (*P* = 1.78 × 10^–5^; type II test), the treatment:genotype interaction does not exert significant impact (*P* = 0.343; type II test) in the context of LNA29 lowering blood cholesterol. In contrast, the treatment:genotype interaction is suggested to be a non-negligible factor (*P* = 0.068; type II test) in the context of LNA29 lowering blood TG. Taken together, these observations suggest that the LNA29-induced lowering of circulating cholesterol is hepatic Sirt1-independent, whereas the LNA29-induced lowering of circulating TG is hepatic Sirt1-dependent.

### Identification of Sirt1-Independent and Sirt1-Dependent Gene/Pathway Changes in the Liver After LNA29 Treatment

In order to characterize the hepatic Sirt1-independent mechanisms that mediate in part the beneficial effects of LNA29, we performed RNA-seq analysis on the liver of L-*Sirt1* KO and control WT/Floxed mice treated with either LNA29 or saline. In WT/Floxed mice, we identified a total of 2397 genes (1254 up; 1143 down) that are altered by LNA29 (Supplementary Figures S2D,E; *P* < 0.05). In L-*Sirt1* KO mice, we identified a total of 1987 genes (1098 up; 889 down) that are altered by LNA29 (Supplementary Figures S2H,I; *P* < 0.05). In theory, miR-29 target genes should be upregulated by LNA29 treatment. By performing miRhub analysis (Baran-Gale et al., 2013), we confirmed that the genes that are upregulated by LNA29 are significantly enriched for predicted target sites of miR-29 in both WT/Floxed (Supplementary Figure S2F) and L-*Sirt1* KO mice (Supplementary Figure S2J), whereas the genes that are downregulated by LNA29 are not in both groups (Supplementary Figures S2G,K).

**FIGURE 3:**
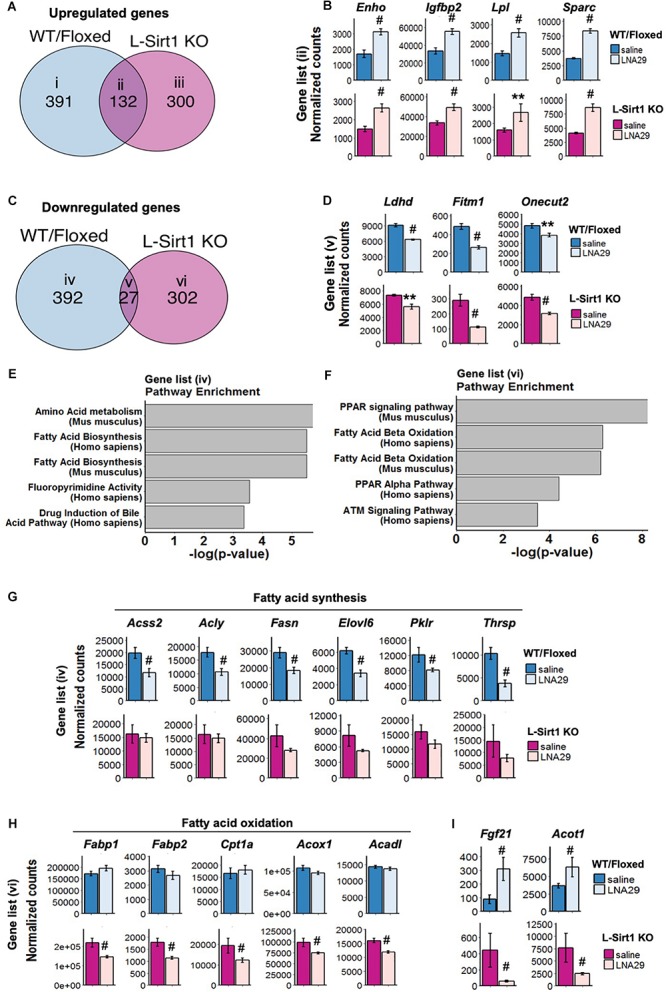
The absence of hepatic Sirt1 blunts the suppression of lipogenesis genes and downregulates fatty acid oxidation (FAO) genes upon LNA29 treatment. **(A)** Venn diagram of upregulated genes that are shared or unique in WT/Floxed and L-*Sirt1* KO mice in response to LNA29 (*P* < 0.05, adjusted *P* < 0.2, base mean >50, fold change>1.2). **(B)** Expressions of upregulated genes shared between WT/Floxed and L-*Sirt1* KO mice in response to LNA29. **(C)** Venn diagram of downregulated genes that are shared or unique in WT/Floxed and L-*Sirt1* KO mice in response to LNA29 (*P* < 0.05, adjusted *P* < 0.2, base mean>50, fold change 1.2). **(D)** Expressions of downregulated genes shared between WT/Floxed and L-*Sirt1* KO mice in response to LNA29. **(E)** Pathway analysis of the downregulated genes unique to WT/Floxed mice in response to LNA29. **(F)** Pathway analysis of the downregulated genes unique to L-*Sirt1* KO mice in response to LNA29. **(G)** Lipogenesis genes are downregulated in WT/Floxed but blunted in L-*Sirt1* KO mice in response to LNA29. **(H)** FAO genes are unchanged in WT/Floxed but downregulated in L-*Sirt1* KO mice in response to LNA29. **(I)** Genes that are changed in opposite direction in WT/Floxed and L-*Sirt1*KO mice in response to LNA29. ^∗∗^*P* < 0.01, ^#^*P* < 0.001 by Wald test (DESeq2) (WT/Floxed-saline, *n* = 5; WT/Floxed-LNA29, *n* = 8; L-Sirt1 KO-saline, *n* = 4; L-Sirt1 KO-LNA29, *n* = 4).

The genes that are altered by LNA29 in both WT/Floxed and L-*Sirt1* KO mice are by definition those that are regulated in a Sirt1-independent manner. By comparing the set of genes that are altered in LNA29-treated WT/Floxed and L-*Sirt1* KO mice (*P* < 0.05, adjusted *P* < 0.2, base mean >50, fold change >1.2 compared to saline) (Figures 3A,C), we identified subsets of genes that are unique to WT/Floxed mice (gene list i in Figure 3A and iv in Figure 3C), unique to L-*Sirt1* KO mice (gene list iii in Figure 3A and vi in Figure 3C), or shared (gene list ii in Figure 3A and v in Figure 3C). The shared genes that are upregulated by LNA29 (gene list ii in Figure 3A) are most enriched in the extracellular matrix (ECM) pathway (Supplementary Figure S3A), which is consistent with the published evidence of a well-established role for miR-29 in regulating ECM genes (Luna et al., 2009; Roderburg et al., 2011). We identified several other shared upregulated genes, such as *Enho*, *Igfbp2*, *Lpl*, and *Sparc* (gene list ii in Figure 3A), that are involved in regulating energy balance and/or metabolism (Figure 3B). In addition, among the small subset of downregulated genes shared between WT/Floxed and L-*Sirt1* KO mice in response to LNA29 (gene list v in Figure 3C and Supplementary Figure S3D) were *Ldhd*, *Fitm1* and *Onecut2* (Figure 3D), which are involved in the process of lipogenesis.

To investigate how the absence of hepatic Sirt1 blunts the LNA29-induced TG lowering effect (Figure 2D), we turned our attention to the genes that are altered by LNA29 only in WT/Floxed or in L-*Sirt1* KO mice but not in both (Figures 3E,F and Supplementary Figures S3B,C,E,F). We found that the genes that are downregulated by LNA29 only in WT/Floxed mice (gene list iv in Figure 3C) are significantly enriched in the fatty acid synthesis pathway (Figure 3E). Several key lipogenic genes, including *Acss2*, *Acly*, *Fasn*, *Elovl6*, *Pklr*, and *Thrsp* (though especially the first two), are significantly downregulated in WT/Floxed but not in L-*Sirt1* KO mice (Figure 3G). In addition, the genes that downregulated by LNA29 only in L-*Sirt1* KO mice (gene list vi in Figure 3C) are significantly enriched in fatty acid oxidation (FAO) pathways (Figure 3F), including *Fabp1*, *Fabp2*, *Cpt1a*, *Acox1*, and *Acadl* (Figure 3H). Taken together, these data show that in the absence of hepatic Sirt1, the inhibition of lipogenic genes is blunted and instead FAO genes are significantly downregulated in response to LNA29 treatment. These findings are consistent with the importance of hepatic Sirt1 in mediating the LNA29-induced TG lowering effect (Figure 2D). Interestingly, we also identified genes that are oppositely affected by LNA29 treatment depending on the Sirt1 genotype (Supplementary Figures S3E,F), which may also contribute to the difference in TG phenotype between LNA29-treated WT/Floxed and L-*Sirt1* KO mice.

### The Effect of LNA29 on Lipogenesis Genes Are Shared With That of BT2 Compound Known to Reduce Liver Fat Partly Through the Suppression of miR-29

**FIGURE 4:**
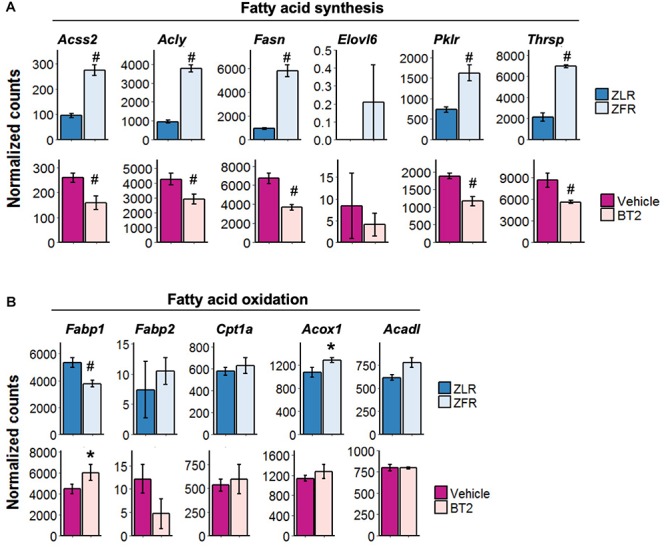
The effect of LNA29 on lipogenesis genes are shared with that of BT2 compound known to reduce liver fat partly through the suppression of miR-29. **(A)** Expressions of lipogenesis genes which are downregulated in LNA29-treated WT/Floxed mice in ZFR compared to ZLR (upper panel) and in ZFR upon BT2 treatment (lower panel). **(B)** Expressions of FAO genes which are downregulated in LNA29-treated L-*Sirt1* KO mice are in ZFR compared to ZLR (upper panel) and in ZFR upon BT2 treatment (lower panel). FAO, fatty acid oxidation, ZFR, Zucker Fatty rat, ZLR, Zucker Lean rat. ^∗^*P* < 0.05 and ^#^*P* < 0.001 by two tailed Students’ *t* test after Bonferroni correction (ZFR, *n* = 4; ZLR, *n* = 4; Vehicle, *n* = 4; BT2, *n* = 4).

The aforementioned RNA-seq analysis of the liver from LNA29-treated L-*Sirt1* KO and WT/Floxed mice suggests that changes in lipogenesis and FAO pathways together determine the LNA29-induced TG lowering phenotype through either Sirt1-dependent or Sirt1-independent manner (Figure 3). We next examined the genes involved in these pathways in an animal model of fatty liver, the ZFR. A recent study described that ZFRs, which develop obesity and NAFLD, have reduced hepatic TG levels upon 1 week treatment with the BT2 compound (inhibitor of branched chain α-keto acid dehydrogenase kinase) (White et al., 2018). We observed elevated miR-29 levels in the liver of ZFRs compared to control ZLRs and a rescue of miR-29 levels in the liver of ZFRs upon BT2 treatment (Supplementary Figures S4A,B), indicating that the reduction of hepatic miR-29 levels is associated with the TG-lowering phenotype of BT2 treatment. We also showed that the same lipogenic genes that are downregulated by LNA29 treatment in a Sirt1-dependent manner (Figure 3G) are significantly upregulated in ZFRs compared to ZLRs and significantly downregulated in ZFRs after BT2 treatment (Figure 4A). Among these lipogenic genes are *Acly*, *Fasn*, *Thrsp*, and *Pklr*, for which protein levels were also significantly reduced as determined by unbiased proteomics analysis (Supplementary Figure S4C). It is worth noting that we found the FAO genes, which are altered by LNA29 only when Sirt1 is absent in the liver (Figure 3H), are not changed much in ZFRs compared to ZLRs or in response to BT2 treatment, with the singular exception of *Fabp1* (Figure 4B). Taken together, these findings suggest that the effects of BT2, a compound shown to ameliorate fatty liver (White et al., 2018), on lipogenic and FAO genes in the ZF rat liver are consistent with the effects of LNA29 on the same pathways in the chow-fed mouse liver. This provides further support to the notion that the TG-improving effect of LNA29 is mediated by the suppression of lipogenic genes.

### LNA29 Treatment Leads to a Global Reduction of TG Species in the Liver Through a Hepatic Sirt1-Dependent Manner

**FIGURE 5:**
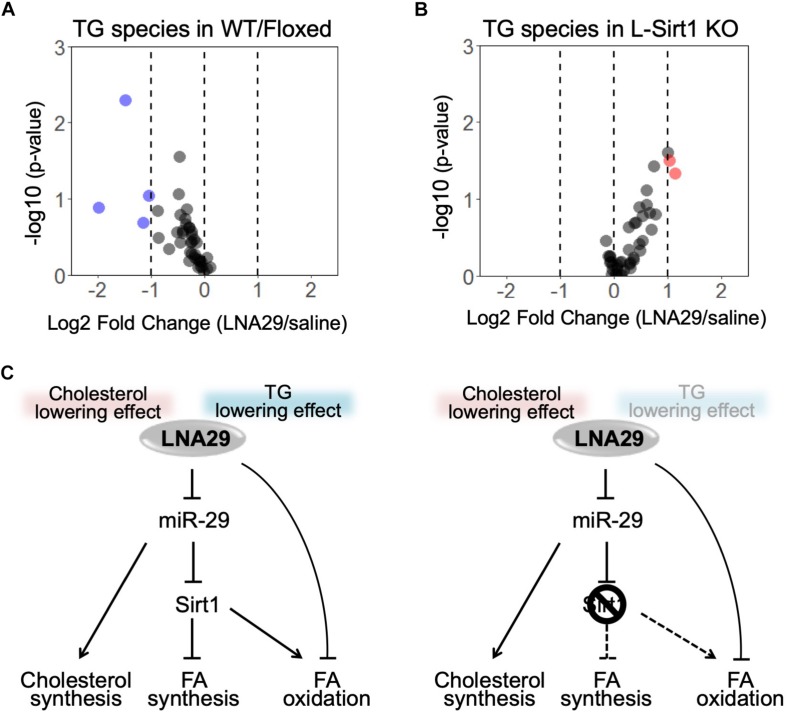
The absence of hepatic Sirt1 blunts LNA29-induced reduction of triglyceride (TG) in the liver. **(A)** Lipidomic analysis of changes in hepatic TG species in WT/Floxed mice with LNA29 compared to saline treatment (saline, *n* = 5; LNA29, *n* = 8). **(B)** Lipidomic analysis of changes in hepatic TG species in L-*Sirt1* KO mice with LNA29 compared to saline treatment (saline, *n* = 5; LNA29, *n* = 5). **(C)** Proposed model of LNA29-mediated effects on hepatic lipid metabolism. The LNA29-mediated cholesterol lowering effect is hepatic Sirt1 independent. The LNA29-mediated TG lowering effect through the suppression of lipogenesis network is hepatic Sirt1 independent. LNA29 leads to downregulation in genes of fatty acid oxidation (FAO) only when hepatic Sirt1 is absent.

Our results suggest that the LNA29-induced changes in genes involved in lipogenesis and FAO depends on the presence of hepatic Sirt1 (Figure 3). Specifically, LNA29-treated WT/Floxed mice exhibit downregulation of genes involved in lipogenesis, whereas LNA29-treatment in L-*Sirt1* KO mice leads to little-to-no changes in lipogenic genes and downregulation of FAO genes (Figure 3). To validate whether the differences we observed at the transcriptomic level between WT/Floxed and L-*Sirt1* KO mice leads to differences in hepatic TG content, we performed lipidomic analysis to profile TG species in the liver of WT/Floxed and L-*Sirt1* KO mice with either saline or LNA29 treatment. We showed that LNA29 treatment leads to a global reduction of TG species in the liver of WT/Floxed mice (Figure 5A, Supplementary Figure S5, and Supplementary Table S1). In contrast, this LNA29-mediated hepatic TG lowering effect is lost in the liver of L-*Sirt1* KO mice (Figure 5B, Supplementary Figure S5, and Supplementary Table S2). Overall, the finding suggests the critical role of hepatic Sirt1 in contributing to the LNA29-mediated TG lowering effect in the liver as well as in the circulation (Figure 5C).

## Discussion

Our previous studies demonstrated that LNA29 treatment improves lipid levels in circulation and highlighted hepatic Sirt1 as a candidate mediator of the effects of LNA29 (Kurtz et al., 2014, 2015). In the present study, we first performed radio-labeled assays to demonstrate that LNA29 treatment leads to suppression of DNL in mouse liver, which likely contributes to the reduction in circulating lipids observed in LNA29-treated mice. Secondly, we evaluated whether hepatic Sirt1 is critical for mediating the beneficial effects of LNA29 using liver-specific Sirt1-deficient (L-*Sirt1* KO) mice. We showed that the LNA29-mediated reduction of liver and circulating TG, but not cholesterol, is in part dependent on hepatic Sirt1. Furthermore, lipidomics analysis revealed a systematic lowering of TG in the liver by LNA29 only in WT but not in L-*Sirt1* KO mice, and comprehensive transcriptomic analysis identified the key fatty acid synthesis genes through which LNA29 may exert these effects.

By performing transcriptome analysis in L-*Sirt1* KO mouse model, we were able to identify Sirt1-independent candidate genes that may mediate some LNA29 effects. For example, *Enho*, *Igfbp2*, *Lpl*, and *Sparc* are upregulated by LNA29 irrespective of the presence of Sirt1 (Figure 3B) and are known to be involved in improving energy homeostasis and/or lipid metabolism (Bradshaw et al., 2003; Wheatcroft et al., 2007; Kumar et al., 2008; Nie et al., 2011; Ganesh Kumar et al., 2012; Chen et al., 2017). Indeed, *Enho*, *Lpl*, and *Sparc* have been shown to be directly targeted by miR-29 (Yang et al., 2016; Song et al., 2018; Hung et al., 2019) and therefore the suppression of miR-29 would be expected to increase the expression and activity of these genes. *Igfbp2*, on the other hand, does not have predicted target sites for miR-29 and its upregulation by LNA29 is possibly through an indirect mechanism. These genes may contribute to some of the LNA29-induced beneficial effects through Sirt1-independent mechanisms. Recently, we reported that LNA29 treatment in WT mice exerts beneficial effects on insulin sensitivity in part through upregulation of hepatic *Enho* (Hung et al., 2019). Enho is also reported to improve cholesterol homeostasis (Ghoshal et al., 2018). In the present study we observed that upon LNA29 treatment L-*Sirt1* KO mice exhibit elevated hepatic *Enho* and reduced circulating cholesterol. Whether Enho plays a critical role in contributing to the effects of LNA29 on cholesterol reduction through a Sirt1-independent manner merits detailed future investigation. In addition, Igfbp2 is known to be involved in the control of circulating cholesterol (Narayanan et al., 2014). Interrogating how this gene may contribute to the reduction of circulating cholesterol in LNA29-treated L-*Sirt1* KO mice will also provide additional insights of Sirt1-independent LNA29 effects.

Transcriptome analysis in the L-*Sirt1* KO mouse model also reveals Sirt1-dependent candidate genes that mediate some of the effects of LNA29, especially as it pertains to TG levels. The data shows that FAO genes respond differently to LNA29 depending on the presence of hepatic Sirt1. Specifically, after LNA29 treatment, we observed little-to-no changes in FAO genes in WT/Floxed control mice but dramatic reduction of FAO genes in L-*Sirt1* KO mice. One hypothesis is that in WT mice hepatic Sirt1 exerts opposing regulatory effects on FAO genes through direct and indirect means, thereby resulting in minimal net changes, and that this balance is disturbed in L-*Sirt1* KO mice (Figure 5C). Furthermore, *Fgf21* and *Acot1* are significantly upregulated in WT/Floxed mice but significantly downregulated in L-*Sirt1* KO mice in response to LNA29 treatment (Figure 3I). Fgf21 has recently emerged as a potential therapeutic target for treating obesity and type 2 diabetes (Coskun et al., 2008; Jimenez et al., 2018). Acot1 has been reported to regulate PPARα-mediated FAO (Dongol et al., 2007). Thus, the downregulation of *Fgf21* and *Acot1* in the absence of hepatic Sirt1 may also diminish the TG lowering effect of LNA29. It is important to note, however, that FAO usually occurs in the fasted state. In this study, we focused on the fed state because our primary focus was lipogenesis. Future work in the fasted state is necessary to dissect the effects of LNA29 on FAO.

It is tempting to consider the potential therapeutic value of LNA29 in the context of fatty liver disease or hyperlipidemia; however, despite the encouraging findings in this study, we issue at least four points of caution and suggestions for next steps. First, it is imperative that more testing of the efficacy of LNA29 in suppressing DNL is performed in animal models of disease. Second, while the delivery method of LNA29 (subcutaneous injection) in this study is very effective for suppressing miR-29 levels in the liver, it is possible that some of the LNA can be taken up by other tissue types as well, albeit likely with reduced efficacy (Kurtz et al., 2015). Thus, it is critical to develop and test tissue-specific LNA delivery mechanisms, and also to expand the examination of miR-29’s biological and molecular functions beyond the liver. Third, miR-29 is known to regulate genes encoding ECM proteins and LNA-mediated suppression of miR-29, especially in hepatic stellate cells, might contribute to the development of hepatic fibrosis (Wang et al., 2015). Careful evaluation of the effects of LNA29 on fibrotic phenotypes is required. Finally, all of the *in vivo* studies conducted thus far have involved single dose, one-week acute response experiments. While single doses of LNA29 appear to be non-toxic (Kurtz et al., 2015), it is unknown what the systemic responses would be to long-term dosing, and this needs to be tested in the future.

Overall, this study demonstrates the beneficial effects of LNA29 on DNL and circulating lipid levels. In addition, this study provides mechanistic insight, using liver-specific *Sirt1*-deficient mice, that decouples the effect of LNA29 on circulating TG from that of circulating cholesterol.

## Data Availability Statement

The datasets generated for this study can be found in the GEO accession GSE129539.

## Ethics Statement

The animal study was reviewed and approved by the Institutional Animal Care and Use Committee (IACUC) of National Health Institutes, NYU  Winthrop Hospital and Duke University.

## Author Contributions

Y-HH contributed to the study design, experiments execution, data collection, and bioinformatic analyses. CK, RC, RB, and LZ carried out the experiments and involved in the data collection. MK performed the bioinformatic analyses. PW, KV, MH, and XL provided resources and animals for critical experiments. PS initiated the project, led the experimental designs, and supervised the study. Y-HH and PS wrote the manuscript. All authors made important intellectual contributions to the experiments and discussion.

## Conflict of Interest

The authors declare that the research was conducted in the absence of any commercial or financial relationships that could be construed as a potential conflict of interest.
