# Supplementary material for: MiR-29 Regulates de novo Lipogenesis in the Liver and Circulating Triglyceride Levels in a Sirt1-Dependent Manner
Source: Front Physiol. 2019 Oct 29;10:1367. doi: 10.3389/fphys.2019.01367 (PMC6828850; doi:10.3389/fphys.2019.01367)
Supplement: Supplementary file 1 [file Table_1.DOCX]

**Supplemental Data**


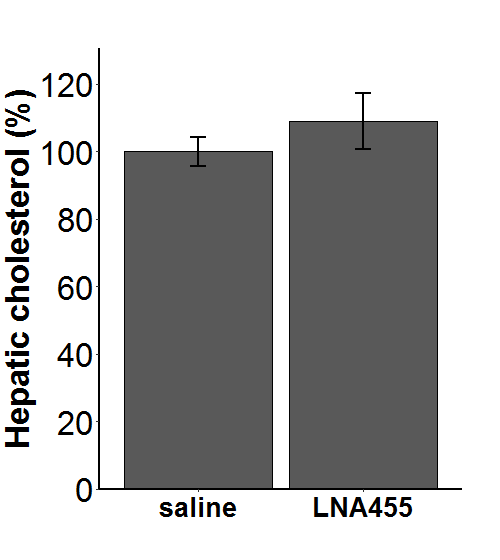

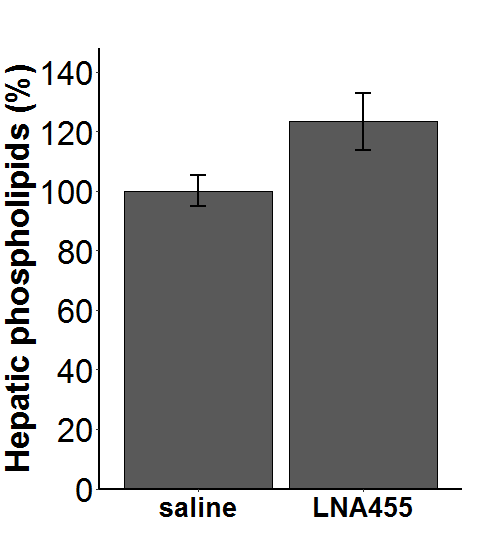

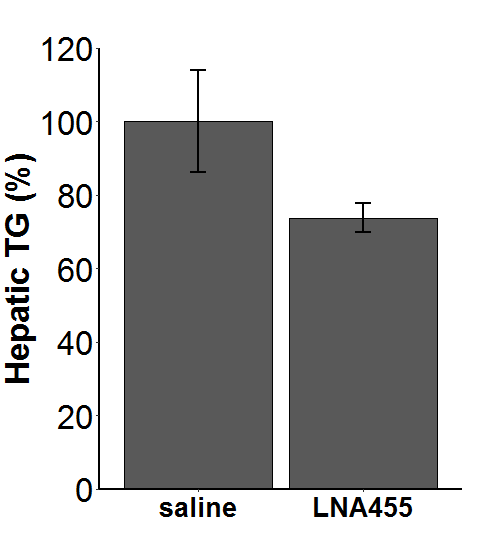


**A**

**B**

P = 0.23

**C**

**Supplemental Figure 1.** Treatment of LNA455 does not alter hepatic lipid levels as well as circulating lipids in chow-fed WT mice. **(A)** Hepatic cholesterol levels of mice treated by either saline or LNA455. **(B)** Hepatic triglyceride levels of mice treated by either saline or LNA455. **(C)** Hepatic phospholipid levels of mice treated by either saline or LNA455. n = 3-6/treatment group.


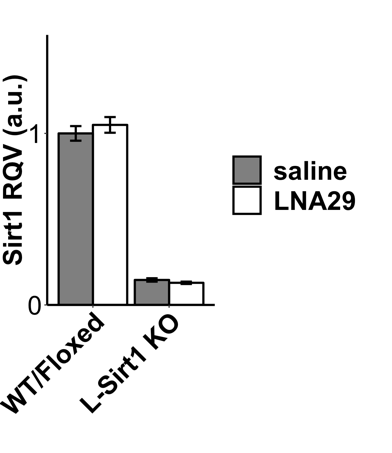

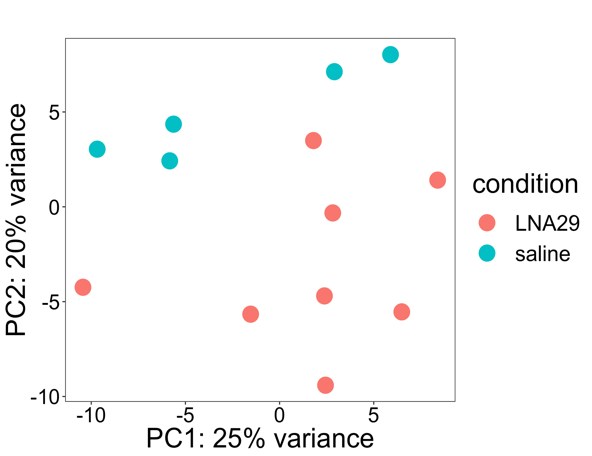

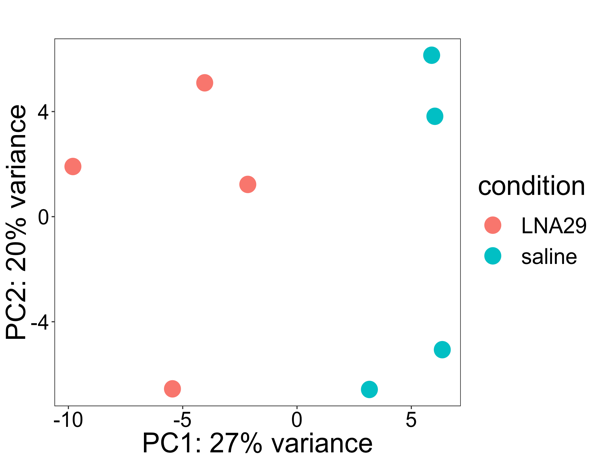


**D**

**G**

**F**

**H**

L-*Sirt1* KO

**I**

**J**

**K**

WT/Floxed


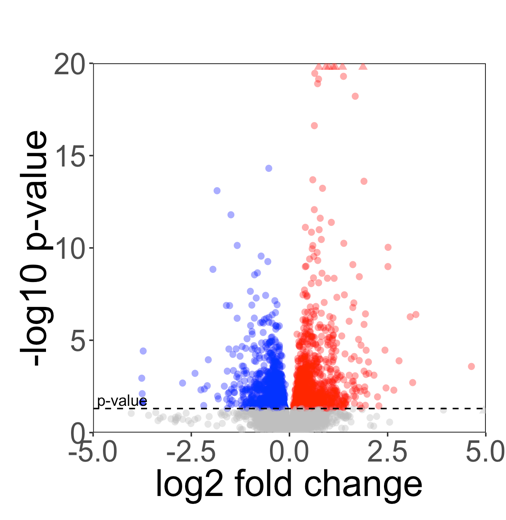


WT/Floxed


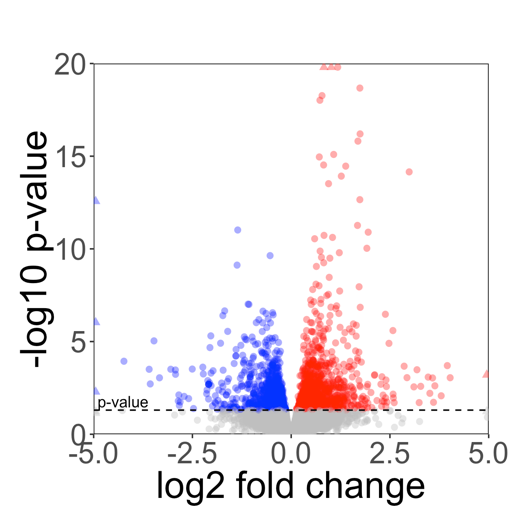


L-*Sirt1* KO

1143 1254

889 1098


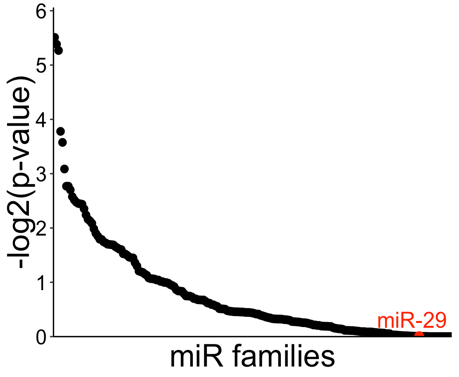

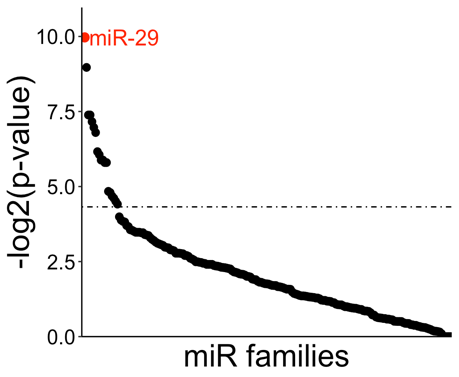


WT/Floxed – upregulated genes

WT/Floxed – downregulated genes


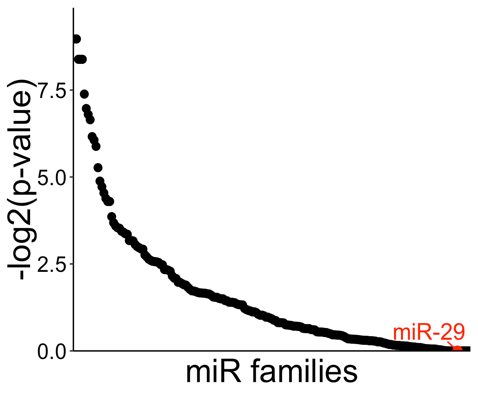

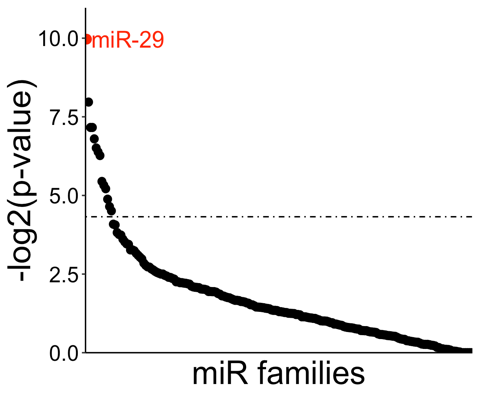


L-*Sirt1* KO –

upregulated genes

L-*Sirt1* KO –

downregulated genes

**E**

*

*


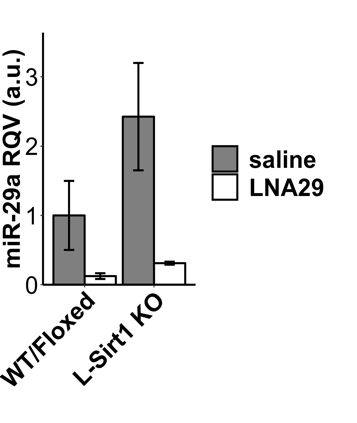

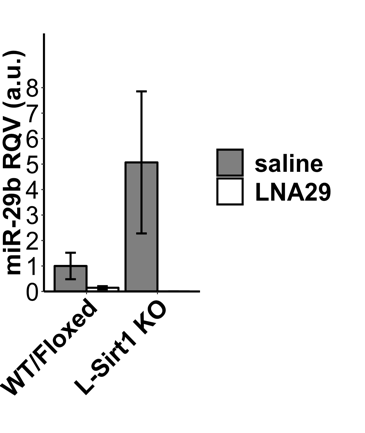

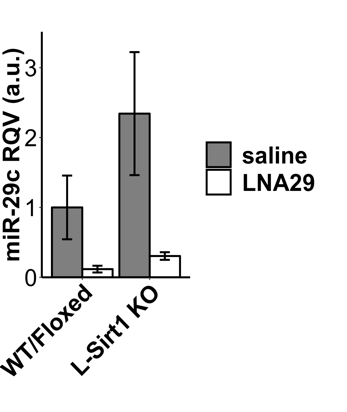


*

*

p = 0.07

p = 0.05


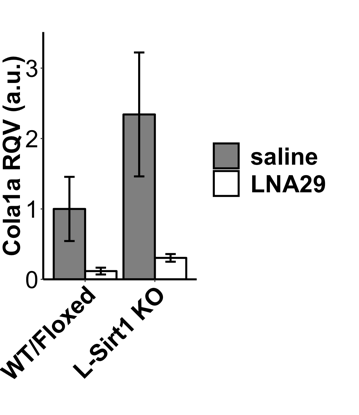


**A**

**B**

**C**

*

p = 0.05

**Supplemental Figure 2.** Transcriptomic profiles of liver from WT/Floxed and L-*Sirt1* KO mice in response to LNA29. **(A)** RT-qPCR of hepatic *Sirt1* in WT/Floxed and L-*Sirt1* KO mice. * P < 0.05 by two-tailed Student’s t-test. **(B)** RT-qPCR of hepatic miR-29 family of WT/Floxed and L-Sirt1 KO mice treated with either saline or LNA29. * P < 0.05 by two-tailed Student’s t-test compared to the saline group within the same genotype. **(C)** RT-qPCR of Co1a1, a validated miR-29 target gene, of WT/Floxed and L-Sirt1 KO mice treated with either saline or LNA29. * P < 0.05 by two-tailed Student’s t-test compared to the saline group within the same genotype. **(D)** PCA plot of RNA-seq profiles from the livers of WT/Floxed mice with saline or LNA29 treatment. **(E)** Volcano plot of differentially expressed genes in the livers of WT/Floxed mice with saline or LNA29 treatment (P < 0.05). **(F-G)** Enrichment analysis of miRNA target sites (using the Monte Carlo simulation tool miRhub) in genes up-regulated (F) and down-regulated (G) in the liver of WT/Floxed mice upon LNA29 treatment (dashed line represents P = 0.05). **(H)** PCA plot of RNA-seq profiles from the livers of L-*Sirt1* KO mice with saline or LNA29 treatment. **(I)** Volcano plot of differentially expressed genes in the livers of L-*Sirt1* KO mice with saline or LNA29 treatment (P < 0.05). **(J-K)** Enrichment analysis of miRNA target sites (using the Monte Carlo simulation tool miRhub) in genes up-regulated (J) and down-regulated (K) in the liver of L-*Sirt1* KO mice upon LNA29 treatment (dashed line represents P = 0.05). WT/Floxed-saline, n = 5; WT/Floxed-LNA29, n= 8; L-Sirt1 KO-saline, n= 4; L-Sirt1 KO-LNA29, n=4.

**
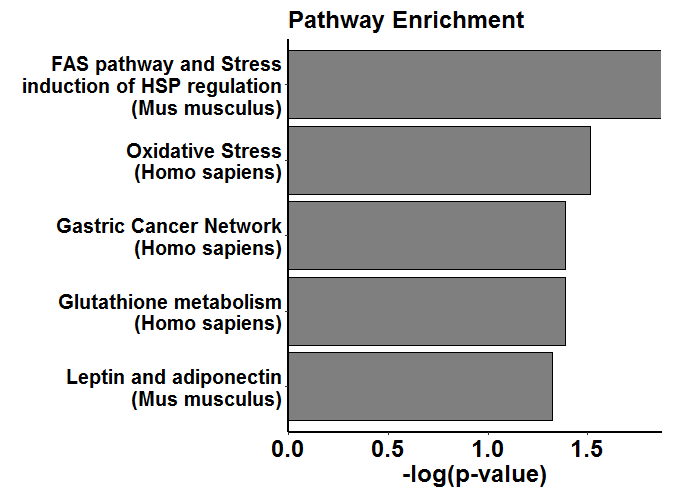

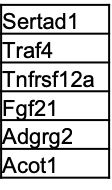

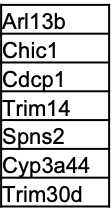
**

**Gene list (v)**

**D**

**Downregulated genes in WT/Floxed**

**Upregulated genes in KO**

**E**

**F**

**Upregulated genes in WT/Floxed**

**Downregulated genes in KO**


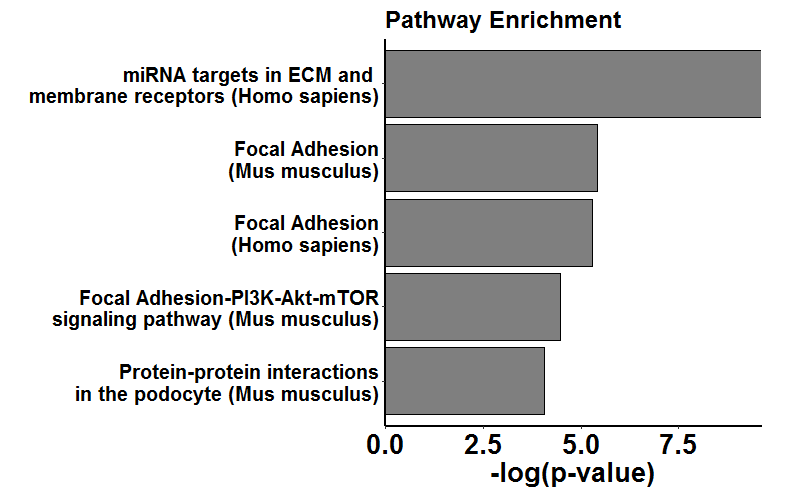


**Gene list (ii)**

**A**

**Gene list (i)**

**B**

**
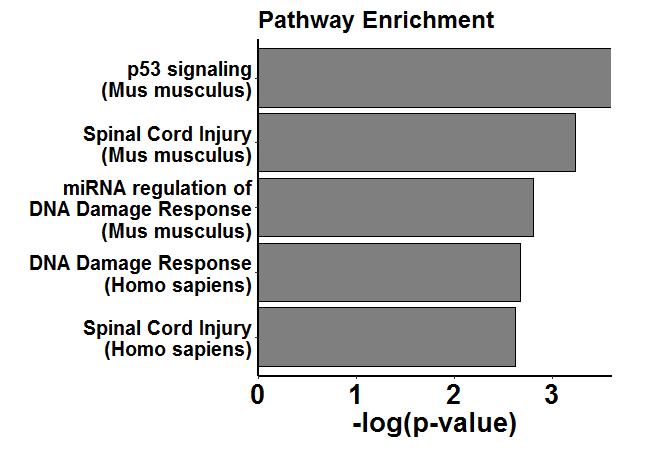
**

**Gene list (iii)**

**C**


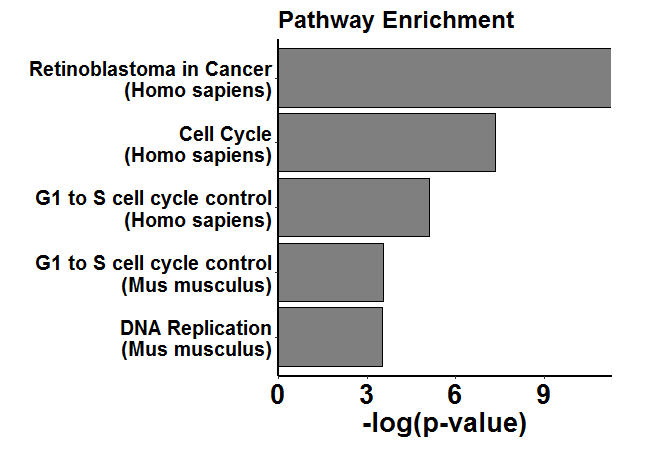


**Supplemental Figure 3.** Glucose metabolism phenotype and pathway analyses of genes shared or unique in WT/Floxed and L-*Sirt1* KO mice in response to LNA29. **(A)** Levels of plasma insulin (left panel) and circulating glucose (right panel) of Floxed mice treated with either saline or LNA29. * P < 0.05 by two-tailed Student’s t-test. **(B)** Pathway analysis of the upregulated genes unique to WT/Floxed mice in response to LNA29. **(C)** Pathway analysis of the upregulated genes unique to L-*Sirt1* KO mice in response to LNA29. **(D)** Pathway analysis of the downregulated genes shared between WT/Floxed and L-*Sirt1* KO mice in response to LNA29. **(E)** Genes that are downregulated in WT/Floxed and upregulated in L-*Sirt1* KO mice. **(F)** Genes that are upregulated in WT/Floxed and downregulated in L-*Sirt1* KO mice. Filtering criteria: P < 0.05 (Wald test), adjusted P < 0.2 (Wald test), base mean > 50, fold change > 1.2 (WT/Floxed-saline, n = 5; WT/Floxed-LNA29, n= 8; L-Sirt1 KO-saline, n= 4; L-Sirt1 KO-LNA29, n=4).

**Supplemental Figure 4.** The effect of LNA29 on lipogenesis genes are shared with that of BT2 compound known to reduce liver fat partly through the suppression of miR-29. **(A)** RT-qPCR of hepatic miR-29 family of ZFR and ZLR. **(B)** RT-qPCR of hepatic miR-29 family of ZFR with either vehicle or BT2 treatment. For (A) and (B), * P < 0.05 and ** P < 0.01 by two tailed Students’ t test (ZFR, n = 4; ZLR, n = 4; Vehicle, n = 4; BT2, n =4). **(C)** Changes in protein levels of lipogenesis genes in ZFR upon BT2 treatment. Data is from the Supplemental Reference (1).


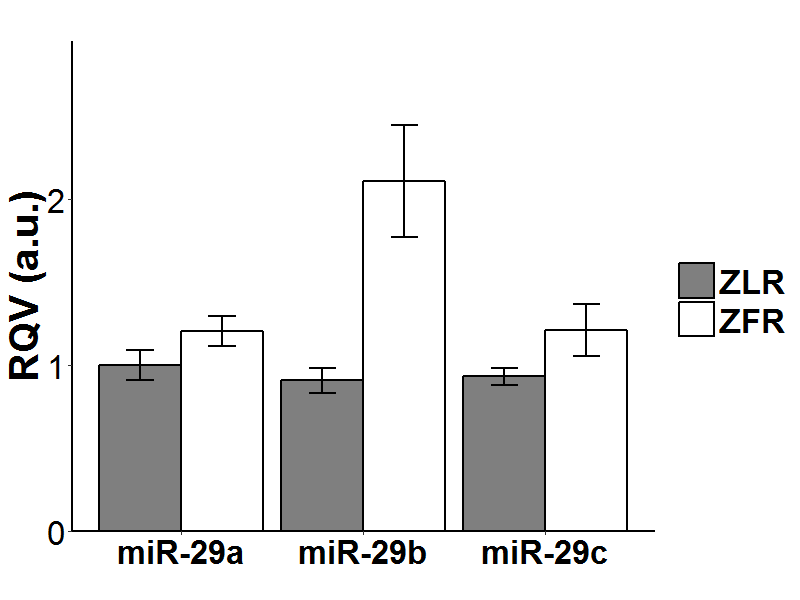


P =0.066

P =0.066

**


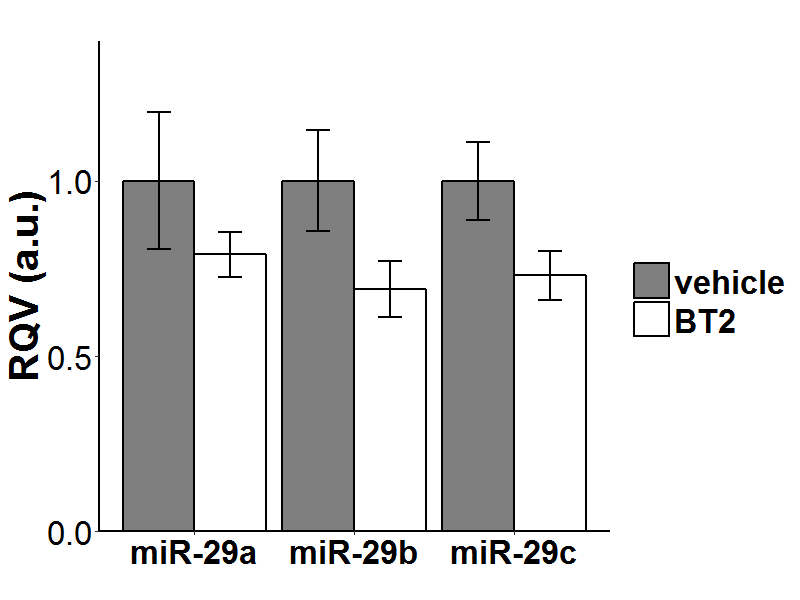


P =0.055

*****

**A**

**B**


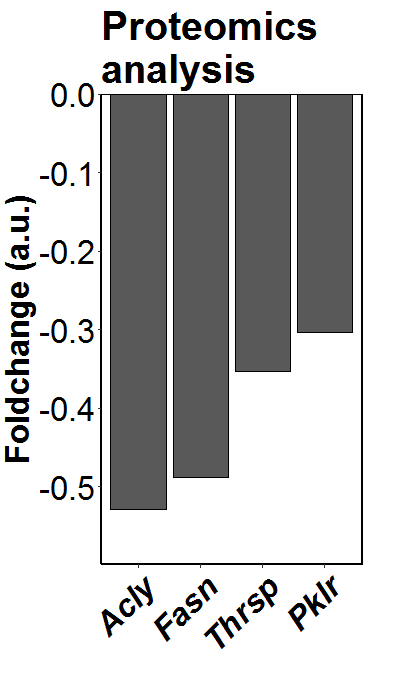


**C**

*

*

*

**P = 0.05**

**B**


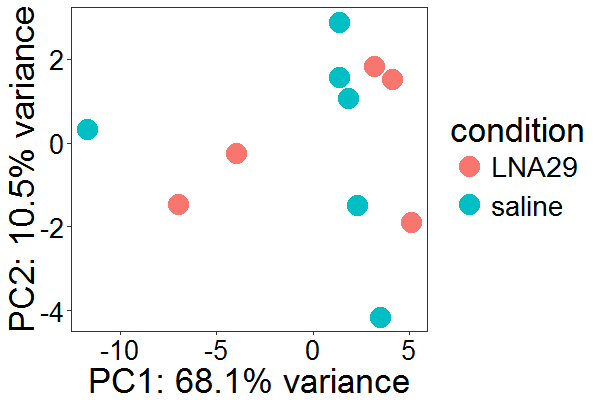

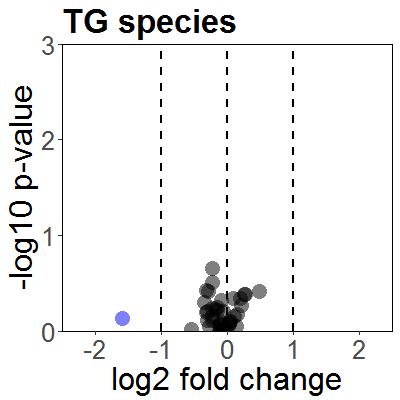


**E**

**F**


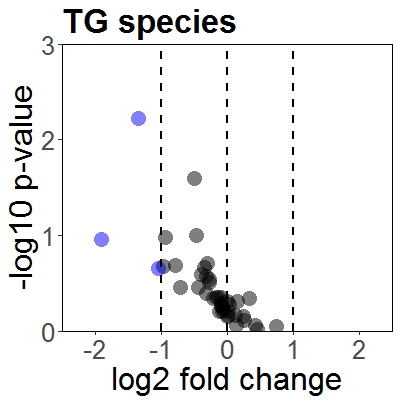

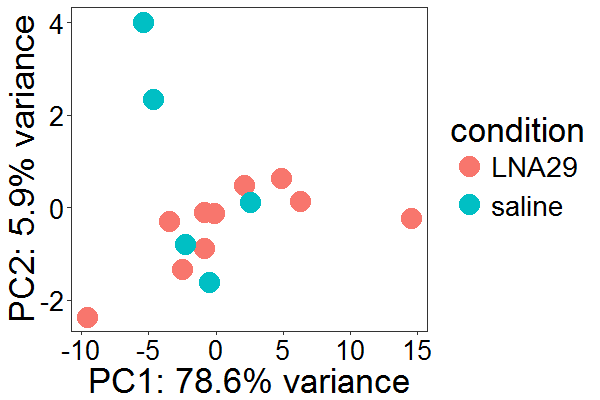


WT/Floxed

L-*Sirt1* KO


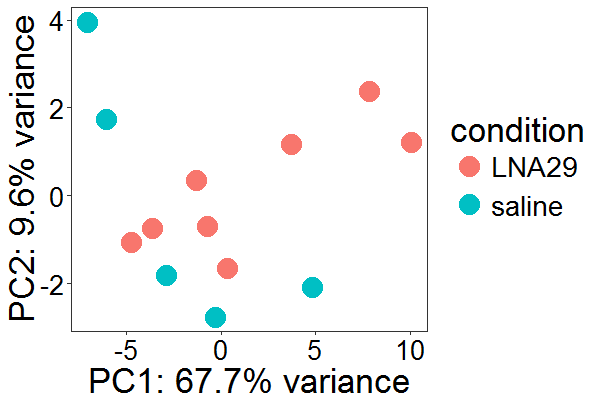

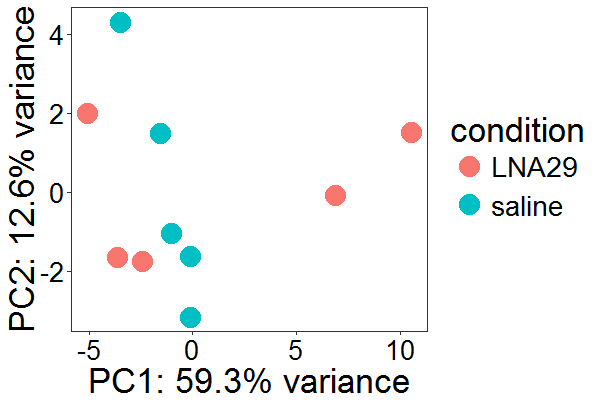


**D**

L-*Sirt1* KO

WT/Floxed

**A**

**C**

**Supplemental Figure 5.** Lipidomic analysis of triglyceride (TG) species in the liver upon LNA29 treatment. **(A)** PCA plot of hepatic TG species in WT/Floxed mice treated with either saline or LNA29 after removal of outlier (indicated in B). **(B)** PCA plot of hepatic TG species in WT/Floxed mice treated with either saline or LNA29 before removal of outliers (indicated by arrows). **(C)** Lipidomic analysis of changes in hepatic TG species in WT/Floxed mice with LNA29 compared to saline treatment before removal of outliers. **(D)** PCA plot of hepatic TG species in L-*Sirt1* KO mice treated with either saline or LNA29 after removal of outlier (indicated in E). **(E)** PCA plot of hepatic TG species in L-*Sirt1* KO mice treated with either saline or LNA29 before removal of outlier (indicated by an arrow). **(F)** Lipidomic analysis of changes in hepatic TG species in L-*Sirt1* KO mice with LNA29 compared to saline treatment before removal of outlier.

**Table 1.** Changes in hepatic TG species in WT/Floxed mice treated with LNA29 compared to saline.

| **TG species** | **Fold Change (FC)** | **log2(FC)** | **p.ajusted** | **p.value** |
| --- | --- | --- | --- | --- |
| TG180/160/160NH4 ISOMER | 1.0819 | 0.11361 | 0.74356 | 0.77588 |
| TG180/160/160NH4 | 1.0475 | 0.066903 | 0.70845 | 0.58379 |
| TG161/182/183NH4 ISOMER | 1.0325 | 0.046097 | 0.94188 | 0.8281 |
| TG161/182/183NH4 | 0.97768 | -0.032563 | 0.9506 | 0.89788 |
| TG161/181/182NH4 | 0.97003 | -0.043894 | 0.92711 | 0.78539 |
| TG161/181/181NH4 ISOMER_1 | 0.96077 | -0.05774 | 0.8808 | 0.65495 |
| TG181/182/182NH4 | 0.94756 | -0.077714 | 0.88422 | 0.77124 |
| TG183/182/183NH4 ISOM-1 | 0.93613 | -0.095213 | 0.88908 | 0.66547 |
| TG160/182/182NH4 | 0.92955 | -0.1054 | 0.88422 | 0.62803 |
| TG160/181/182NH4 ISOM | 0.92553 | -0.11164 | 0.86677 | 0.77445 |
| TG161/181/181NH4 | 0.91703 | -0.12496 | 0.8808 | 0.61458 |
| TG161/181/182NH4 ISOMER | 0.91455 | -0.12886 | 0.70845 | 0.36765 |
| TG200/181/181NH4 | 0.89467 | -0.16058 | 0.74356 | 0.53607 |
| TG181/181/181NH4 ISOM-2 | 0.89313 | -0.16305 | 0.70845 | 0.57852 |
| TG181/181/204NH4 | 0.87524 | -0.19225 | 0.70845 | 0.33816 |
| TG181/181/181NH4 ISOM-1 | 0.85278 | -0.22976 | 0.63512 | 0.29294 |
| TG191/181/181NH4 ISOM-1 | 0.85199 | -0.23109 | 0.66747 | 0.3586 |
| TG182/182/182NH4 ISOM-3 | 0.84285 | -0.24665 | 0.70845 | 0.26191 |
| TG182/182/182NH4 ISOM-2 | 0.83901 | -0.25324 | 0.74356 | 0.51798 |
| TG181/181/182NH4 ISOM-1 | 0.83247 | -0.26453 | 0.65139 | 0.37491 |
| TG191/181/181NH4 | 0.82848 | -0.27145 | 0.65139 | 0.48913 |
| TG181/181/211NH4 | 0.82359 | -0.28 | 0.63194 | 0.65116 |
| TG182/182/182NH4 ISOM-1 | 0.82321 | -0.28066 | 0.78626 | 0.23302 |
| TG181/181/225NH4 ISOM-1 | 0.82019 | -0.28597 | 0.63512 | 0.23621 |
| TG201/181/181NH4 | 0.80076 | -0.32055 | 0.55968 | 0.13493 |
| TG181/181/182NH4 | 0.78376 | -0.35152 | 0.63512 | 0.21334 |
| TG181/181/225NH4 | 0.7764 | -0.36512 | 0.63194 | 0.18141 |
| TG180/180/200NH4 ISOMER | 0.76263 | -0.39094 | 0.63194 | 0.25923 |
| TG182/182/182NH4 | 0.74416 | -0.42631 | 0.65139 | 0.2784 |
| TG181/181/211NH4 ISOM-1 | 0.72941 | -0.45519 | 0.55968 | 0.37303 |
| TG181/181/181NH4 | 0.72651 | -0.46095 | 0.36796 | 0.15917 |
| TG200/181/181NH4 ISOM-1 | 0.7226 | -0.46874 | 0.55968 | 0.027782 |
| TG183/182/183NH4 | 0.72152 | -0.47089 | 0.65139 | 0.086622 |
| TG201/181/181NH4 ISOM-1 | 0.71055 | -0.49299 | 0.55968 | 0.27111 |
| TG181/182/182NH4 ISOM-1 | 0.63375 | -0.65802 | 0.63194 | 0.44968 |
| TG161/181/181NH4 ISOMER_2 | 0.55566 | -0.84772 | 0.63194 | 0.32219 |
| TG160/181/182NH4 | 0.55416 | -0.85161 | 0.55968 | 0.14145 |
| TG180/180/200NH4 | 0.48559 | -1.0422 | 0.55968 | 0.090686 |
| TG181/181/182NH4 ISOM-2 | 0.45431 | -1.1382 | 0.55968 | 0.20374 |
| TG181/181/204NH4 ISOM-1 | 0.36644 | -1.4483 | 0.023308 | 0.0050803 |
| TG160/182/182NH4 ISOMER | 0.25469 | -1.9732 | 0.55968 | 0.12897 |

Note: TG161/182/183 represent a TG molecule with fatty acids C16:1 (position 1), C18:2 (position 2) and C18:3 (position 3).

**Table 2.** Changes in hepatic TG species in L-Sirt1 KO mice treated with LNA29 compared to saline.

| TG species | Fold Change (FC) | log2(FC) | p.ajusted | p.value |
| --- | --- | --- | --- | --- |
| TG161/182/183NH4 ISOMER | 2.206 | 1.1415 | 0.60208 | 0.046339 |
| TG183/182/183NH4 | 2.0509 | 1.0363 | 0.60208 | 0.031477 |
| TG183/182/183NH4 ISOM-1 | 1.989 | 0.99203 | 0.60208 | 0.02488 |
| TG161/182/183NH4 | 1.7113 | 0.77507 | 0.60208 | 0.15495 |
| TG182/182/182NH4 ISOM-3 | 1.673 | 0.74244 | 0.60208 | 0.037286 |
| TG160/181/182NH4 | 1.616 | 0.69241 | 0.77267 | 0.24792 |
| TG182/182/182NH4 ISOM-2 | 1.5811 | 0.66089 | 0.60208 | 0.15015 |
| TG160/182/182NH4 | 1.5287 | 0.61234 | 0.60208 | 0.11852 |
| TG181/181/204NH4 | 1.5142 | 0.59857 | 0.60208 | 0.075373 |
| TG182/182/182NH4 ISOM-1 | 1.4509 | 0.53692 | 0.71069 | 0.347 |
| TG161/181/182NH4 ISOMER | 1.4498 | 0.53581 | 0.60208 | 0.16239 |
| TG182/182/182NH4 | 1.3986 | 0.48396 | 0.77267 | 0.46305 |
| TG161/181/182NH4 | 1.38 | 0.46467 | 0.60208 | 0.12737 |
| TG161/181/181NH4 | 1.3789 | 0.46351 | 0.77267 | 0.37778 |
| TG181/181/225NH4 | 1.332 | 0.41358 | 0.60208 | 0.19995 |
| TG181/181/225NH4 ISOM-1 | 1.3033 | 0.38219 | 0.60208 | 0.20571 |
| TG200/181/181NH4 | 1.2841 | 0.36075 | 0.91512 | 0.57338 |
| TG160/181/182NH4 ISOM | 1.2824 | 0.3588 | 0.91512 | 0.60744 |
| TG160/182/182NH4 ISOMER | 1.2342 | 0.30363 | 0.91512 | 0.65246 |
| TG181/181/211NH4 ISOM-1 | 1.2315 | 0.30041 | 0.91512 | 0.7813 |
| TG161/181/181NH4 ISOMER_2 | 1.219 | 0.28575 | 0.91512 | 0.68452 |
| TG161/181/181NH4 ISOMER_1 | 1.2051 | 0.26915 | 0.66468 | 0.2299 |
| TG181/182/182NH4 | 1.2031 | 0.26673 | 0.89502 | 0.44801 |
| TG181/181/182NH4 ISOM-2 | 1.1388 | 0.18746 | 0.945 | 0.83307 |
| TG181/181/204NH4 ISOM-1 | 1.0956 | 0.13176 | 0.945 | 0.63874 |
| TG191/181/181NH4 | 1.0954 | 0.13146 | 0.945 | 0.95421 |
| TG201/181/181NH4 ISOM-1 | 1.0852 | 0.11795 | 0.945 | 0.98062 |
| TG181/181/182NH4 ISOM-1 | 1.0751 | 0.10445 | 0.945 | 0.66445 |
| TG180/180/200NH4 | 1.047 | 0.066321 | 0.945 | 0.79 |
| TG180/160/160NH4 ISOMER | 1.0385 | 0.054552 | 0.945 | 0.70753 |
| TG181/181/182NH4 | 1.0299 | 0.042534 | 0.945 | 0.94932 |
| TG181/182/182NH4 ISOM-1 | 1.0176 | 0.025183 | 0.945 | 0.91951 |
| TG181/181/211NH4 | 0.98181 | -0.026483 | 0.945 | 0.82786 |
| TG180/160/160NH4 | 0.98112 | -0.027501 | 0.945 | 0.71647 |
| TG180/180/200NH4 ISOMER | 0.97089 | -0.042616 | 0.945 | 0.94061 |
| TG181/181/181NH4 ISOM-2 | 0.96303 | -0.05435 | 0.945 | 0.64418 |
| TG191/181/181NH4 ISOM-1 | 0.95353 | -0.06865 | 0.945 | 0.66142 |
| TG201/181/181NH4 | 0.94964 | -0.07455 | 0.945 | 0.547 |
| TG181/181/181NH4 | 0.94815 | -0.076818 | 0.945 | 0.55464 |
| TG181/181/181NH4 ISOM-1 | 0.93007 | -0.10458 | 0.945 | 0.545 |
| TG200/181/181NH4 ISOM-1 | 0.89676 | -0.15721 | 0.91512 | 0.34911 |

Note: TG161/182/183 represent a TG molecule with fatty acids C16:1 (position 1), C18:2 (position 2) and C18:3 (position 3).

**Supplemental reference:**

1. White PJ, McGarrah RW, Grimsrud PA, Tso SC, Yang WH, Haldeman JM, et al. The BCKDH Kinase and Phosphatase Integrate BCAA and Lipid Metabolism via Regulation of ATP-Citrate Lyase. Cell Metab. 2018;27(6):1281-93 e7.
